# Prevalence of nail biting and its association with mental health in a community sample of children

**DOI:** 10.1186/1756-0500-4-116

**Published:** 2011-04-11

**Authors:** Ahmad Ghanizadeh, Hajar Shekoohi

**Affiliations:** 1 Research Center for Psychiatry and Behavioral Sciences, Hafez Hospital, Shiraz, Iran; 2 Department of Psychiatry, Shiraz University of Medical Sciences, Hafez Hospital, Shiraz, Iran

## Abstract

**Background:**

This study evaluates onychophagia or nail biting (NB) prevalence and association with mental health of a community sample of children from Shiraz, Iran.

**Findings:**

The parents of 743 primary school children, selected by random sampling, reported NB behavior of their children and themselves. Children's mental health problem was assessed using the Strengths and Difficulties Questionnaire (SDQ). 22.3% (95% CI: 19.3 to 25.3) of children had NB behavior in the last three months (girls: 20.1% (95% CI: 15.9 to 24.2). The rate in boys was 24.4% (95% CI: 20.1 to 28.7). 36.8% of the children with NB had at least one family member with nail biting. Older age was associated with a higher prevalence of NB while a higher score on the prosocial score was associated with a lower prevalence of NB.

**Conclusions:**

NB is a very common behavior in both genders in children and their family members. Children with NB have less prosocial ability than those without it.

## Findings

Nail biting (NB) or onychophagia is a common but unresolved medical problem in children [1]. A study on 248 girls aged 15-16 years reported the rate of 25.5% [2]. Another study on 385 school children aged 12-16 years using a questionnaire reported the rate of 29%[3].

Its incidence in the ages of 4-6 years is more than earlier ages. Its rate increases in adolescence while it declines in later ages [1]. NB is not gender dependent in children less than 10 years but its incidence in boys is more than girls in adolescents [1].

NB may induce complications such as malocclusion of the anterior teeth [1], teeth root resorption [4], intestinal parasitic infections [5], change of oral carriage of enterobacteriaceae [6], bacterial infection, and alveolar destruction [1]. Moreover, about one forth of patients with temporo-mandibular joint pain and dysfunction have nail biting habit [7].

Given that the treatment of nail biting is not easy. Many of children with NB and their parents have already tried to stop it but they were not successful. May parents may punish or ridicule or even pressure the children to stop it. But these attempts are not usually effective in long term. Probably, the studying of associated factors to NB helps us for planning more effective managements [8].

At least, in clinical samples, NB reflects underlying emotional disorders [9]. A study on clinical sample of children showed that boys with NB suffer from at least one of the psychiatric disorders more than girls that most likely were attention deficit hyperactivity disorder (ADHD) 0.6%, oppositional defiant disorder 36%, and separation anxiety disorder 20.6%. The presence of psychiatric disorders is not associated with the age of onset, the frequency of NB, and the existence of physical damage [9]. NB is one of the most common behavioral problems (228.6%) in children and adolescents with Tourette syndrome [10]. Its rate in children with ADHD is about 40.0% [11,12]. 14.3% of children with alopecia areata suffer from NB [13]. It is suggested that NB as a behavioral sensory processing problem may help us for a better pharmacological management of children with ADHD [14].

Moreover, more than half of parents of children with NB have at least one psychiatric disorder most likely it is major depression [9]. However, that study mentioned that their sample was not typical of children with NB in the general population. It recommended that future studies including community samples should be conducted.

The current cross-sectional study aimed to survey prevalence of nail biting in school aged children. The investigation of possible associating of NB with emotional and behavioral problems is another aim of this study.

## Materials and methods

The participants of this study are the parents of one thousand school children selected by randomized cluster sampling. 743 completed questionnaires were returned by the parents. 358(48.2%) were boys and 385 were girls.

The parents reported their children's nail biting behavior and his/her emotional and behavioral problems. Parents reported the numbers of days per week whereby the children bite their nail [9]. Parents were also questioned whether there is anybody else with nail biting behavior in their family.

### Strengths and Difficulties Questionnaire (SDQ)

The Strengths and Difficulties Questionnaire (SDQ) is a research tool with adequate validity and reliability. It is consisted of 25 items including five clinical scales: hyperactivity/inattention, emotional symptoms, conduct problems, peer relationship problems, and prosocial behavior. Each of the clinical scales has 5 items. Response to each item can range from 0 to 2. Zero was for ''not true", 1 for ''somewhat true'', and 2 for ''certainly true". Positive items are reverse coded except for prosocial scale. So, the score of each clinical scale can range from 0 to 10. Summing the scores from the scales of hyperactivity/inattention, emotional symptoms, conduct problems, and peer relationship problems generates a total difficulties score. The resultant score can range from 0 to 40. Therefore, the prosocial scale is not included for generating of total score. The parent version of SDQ was used here. The psychometric properties of its Farsi version has been studied before [15]. SDQ takes about 5 to 10 minutes to be completed by parents. Its scoring system is clear and straightforward. Contrary to the other subscales of SDQ, higher score of prosocial subscale indicates a better condition. SDQ is a valuable brief screening instrument for the adjustment and psychopathology of children [16,17]. The SDQ, its Farsi language translation, scoring information, and publications list can be retrieved from the Web site of http://www.sdqinfo.com.

Eight primary schools in the different geographic locations in the city of Shiraz, southern of Iran, were selected by stratified random sampling method. All of the principals of the schools agreed for participation in the study after they were informed. They permitted us to contact the parents. The questionnaire was delivered to the parent by the students and then they completed and returned back. Information about the aim of this study was provided to the children and their parents. Participation in this study was voluntary. The Good Clinical Practice Guidelines, in accordance with the Declaration of Helsinki, 1975, as revised in 2000 was considered for conducting of this study. Parental consent was provided by the parents of the children involved in the study. This study was approved by Ethics Committee of Shiraz University of Medical Sciences.

### Statistical analysis

Statistical analysis was conducted using SPSS statistical package. The frequency of nail biting by children and their family members were analyzed for boys and girls. t-test was used to compare the mean age of boys and girls. Chi-Square test was used to study the association of nail biting behavior and gender.

Logistic regression analysis was conducted to examine association of the predictable factors and nail biting. Nail biting was considered as a categorical variable. The response categories of "never" and "I days per week" was coded as zero and all other categories (i.e. >= 2 days per week) were coded as 1. The variables of age, parental educational level, rank of birth, prosocial score, emotional score, hyperactivity/inattention score, conduct problem score, and peer problem score were considered as independent factors. The educational levels of parents were considered as continues variables.

## Results

The mean age of the sample was 8.2(SD = 0.8) years with the age range of 7 to 10 years. The mean age of boys and girls was not different (t = 1.1, df = 741, P = 0.2). 358(48.25) of the sample were boys. 41.0% of the children were the first child of their family. 28.4% were the second child of family. 21.7% of the whole sample was the only child. 38.4% had at least one brother or sister.

The rate of nail biting in boys and girls was 20.1% (95% confidence interval 15.9 to 24.2) and 24.4% (95% confidence interval 20.1 to 28.7), respectively. The rate of nail biting was not statistically different between boys and girls (X2 = 1.98, df = 1, P = 0.1) (Table 1).

**Table 1:** Frequency of parent reported nail biting.

Frequency	Boys		Girls		Total		Significancy
		
	Number	Percent	Number	Percent	Number	Percent	
Always (4 days or more per week)	18	5.0	19	4.9	37	5.0%	X^2 ^= 2.6, df = 3, P = 0.4
sometimes (2-3 days per week)	21	5.9	32	8.3	53	7.1%	
Rare (1 day per week)	33	9.2	43	11.2	76	10.2%	
Never	286	79.9	291	75.6	577	77.7%	

577(77.7%) of the parents reported that their children have not habitually bitted nail in the last three months. 5.0% reported that their child frequently (more than 3 days per week) bitted his/her nail in the last three months. The rate for children with 2-3 days and 1 day were 7.1% and 10.2%, respectively. So, 22.3% of the sample had the habit of nail biting with frequency of at least a day in week in the last three months. Out of the 166 children with nail biting, 45.8% of them bitted it 1 day per week. The frequency of nail biting per day is indicated in Table 2.

**Table 2:** The frequency of nail biting per day.

Times per day	Frequency	Percent
1 to 2	97	58.4
3 to 4	34	20.5
5 to 6	8	4.8
More than 6	12	7.2
Total	151	91.0

The discrepancy between total number and the number of children with nail biting is due to missing data.

36.8% (95% CI: 22.3 to 44.2) of the children with nail biting had at least one family member with nail biting. 22.9% of children with nail biting have at least one brother or sister with nail biting (Table 3). The parents of 13.2% of children with nail biting habitually bite their nail (Table 3). From 93 children with NB who has at least one sibling, 31(33.3%) had a sibling with NB. Totally, 53(55.8%) of children with NB who has at least one sibling, at least one of their siblings or parents bite his/her nail frequently (Table 4).

**Table 3:** The frequency of nail biting in the siblings or parents of children with nail biting.

Family member	Frequency	Percent
Sister	17	10.2
Brother	21	12.7
Father	8	4.8
Mother	14	8.4
Nobody	103	62.0

**Table 4:** The prevalence of nail biting in family members of children with NB who has at last one sibling (n = 93)

Family member	Frequency	Percent
Sister	14	14.7
Brother	17	17.9
Father	2	2.1
Mother	7	7.4
Total	53	55.8

Table 5 indicates the mean scores of different domains of parent reported SDQ for children with NB and those without NB. It displays that the scores of prosocial behavior score, emotional score and conduct score are significantly different between children with and those without nail biting. Prosocial behavior score in children with nail biting is less than that those counterparts without nail biting. Emotional problem score and conduct problem score of children with nail biting are more than that of those children without nail biting.

**Table 5:** The comparison of the mean score of different domains of parent reported SDQ between children with nail biting (> = 1 day per week) and that of those without nail biting.

Scale	Nail biting	Mean	Standard Deviation	95% Confidence Interval for Mean	
				
				Lower Bound	Upper Bound
Prosocial behavior score	Without	8.15	1.6	8.0	8.3
	With	7.6	2.0	7.2	7.9
Hyperactivity/inattentiveness scale	Without	5.5	1.6	5.4	5.7
	With	5.8	1.7	5.5	6.0
emotional problem score	Without	3.7	1.8	3.5	3.8
	With	4.3	2.1	4.0	4.6
Conduct problem Score	Without	2.9	1.4	2.8	3.0
	With	3.2	1.4	3.0	3.4
Peer problem score	Without	4.9	1.5	4.8	5.1
	With	5.1	1.6	4.8	5.4
Total score	Without	15.9	4.7	15.4	16.4
	With	17.3	4.6	16.5	18.1

Logistic regression analysis indicated that the factors of age of children and prosocial score have a statistically significant association with nail biting frequency in children. None of the parental educational level, gender, rank of birth, hyperactivity/inattention score, conduct problem score, and peer problem score significantly predicts nail biting frequency in children. There was a marked trend for the association of emotional problems and nail biting (Table 6).

**Table 6:** Logistic regression analysis for the predictors of parent-reported frequency of nail biting in children

	Odds ratio	95% C.I.		Significance
			
		Lower	Upper	
Age	1.5	1.12	2.07	.006
Gender	1.4	.81	2.44	.22
Rank of birth	.8	.65	1.17	.36
Mother's educational level	1.0	.90	1.13	.77
Father's educational level	1.0	.99	1.19	.07
Prosocial behavior score	.8	.75	.99	.044
Hyperactivity/inattention scale	.9	.83	1.15	.81
emotional problem score	1.1	.98	1.32	.07
Conduct problem Score	1.1	.95	1.39	.14
Peer problem score	1.0	.85	1.20	.87

## Discussion

The most striking findings of our study are that emotional and behavioral problems are more common in children with nail biting than those without nail biting. More than one fifth of children have this habit. In addition, our results added to literature that NB has a family pattern. There was not a significant association between nail biting and emotional problems, however, children with NB have higher rates of emotional problems than children without NB. In addition, the prosocial behavior of children with NB is weaker than that of those without NB. This behavior is age dependent but it is not gender related.

Nail-biting allows children relieve their anxiety, loneliness, and deprivation of safety feeling and love [18]. However, there is a controversy about the association of anxiety, stress and NB [19,20]. In the current study, emotional problems are higher in those with NB. It should be noticed that emotional problems are not equal to anxiety. In addition, possible cause and effect of anxiety and NB has not already been reported. Meanwhile, 20% of clinical sample of children with NB had separation anxiety disorder [9]. It is possible that both of them are secondary to a common factor. Moreover, NB is a common co-existing problem in many psychiatric disorders [10,21,22] and these co-morbidities may be an explanation for the higher rate of emotional problems in children with NB. Another possible explanation for the higher rate of emotional problems in children with NB may be due to the consequences of NB. Social and family pressure for stopping this behavior may induce or increase emotional problems of these children. Maybe these children will experience higher level of emotional problems in later ages.

Children with NB especially boys had lower score of prosocial behavior score. This scale indicates social relationship ability. So, the lower score of prosocial behavior score is related to lower ability. This lower ability may be another explanation for higher emotional problems in children with NB.

The mean score for conduct problem in children with NB was more than that of those without NB. But after considering the covariant factors, conduct problems score did not predicted nail biting behavior. The association of NB and aggression has been reported before [23]. However, they did not include covariant factor.

The present study as some previous study has shown that the prevalence of parent reported NB is not gender associated [1]. It is clear that this study is a cross sectional study and it investigates the association of NB and children's emotional and behavioral problems.

There are several research and clinical implications for these findings. The research implication is that the common behavior of nail biting should be studies more, although research and evidence on nail biting etiology and its associations are becoming more available. One of the clinical implications is that nail biting occurs frequently in other members of family. So, the questioning about nail biting in children and students can be considered as a screening question. Positive response may indicate high probability for happening in other members of family. So, child psychiatric nurses and school nurses should be very careful about this behavior. They should evaluate children with nail biting for screening of emotional problems and less developed prosocial behaviors. Another clinical implication is that the other family members of children with nail biting should be evaluated and screened for nail biting. Psychological evaluations should not be limited on the children. So, it seems that nail biting should not be considered as a simple habit. There are many consequences for nail biting that should be considered. For example, these children may require to be referred for dentistry assessment. Our finding has an important clinical implication for management of these children. Habit reversal which is usually used for their management is not effective in long term in many cases. An explanation for this failure can be due to lack of consideration of co-occurring emotional and behavioral problems.

In conclusion, nail biting is a very common, age dependent, but not gender related behavior in school age children. NB is associated with lower prosocial skills in the community sample of children.

## Competing interests

The authors declare that they have no competing interests.

## Authors' contributions

AG: Research project conception and organization. Statistical analysis design, execution, review and critique. Writing the first draft and review and critique of the manuscript. HS: Research project organization and execution. Statistical analysis execution. Writing the first draft and review and critique of the manuscript. All authors read and approved the final manuscript.
